# Radionuclide Tracing Based in situ Corrosion and Mass Transport Monitoring of 316L Stainless Steel in a Molten Salt Closed Loop

**DOI:** 10.21203/rs.3.rs-3415493/v1

**Published:** 2023-10-09

**Authors:** Yafei Wang, Aeli Olson, Cody Falconer, Brian Kelleher, Ivan Mitchell, Hongliang Zhang, Kumar Sridharan, Jonathan Engle, Adrien Couet

**Affiliations:** University of Wisconsin-Madison; University of Wisconsin-Madison; University of Wisconsin-Madison; TerraPower, LLC; TerraPower, LLC; Fudan University; University of Wisconsin-Madison; University of Wisconsin - Madison; University of Wisconsin-Madison

## Abstract

In the study, we report an *in situ* corrosion and mass transport monitoring method developed using a radionuclide tracing technique for the corrosion study of 316L stainless steel (316L SS) in a NaCl-MgCl_2_ eutectic molten salt natural circulation loop. This novel method involved cyclotron irradiation of a small tube section with 16 MeV protons, later welded at the hot leg of the molten salt flow loop, generating radionuclides ${\;}^{51}\text{Cr}$, ${\;}^{52}\text{Mn}$, and ${\;}^{56}\text{Co}$ at the salt-alloy interface. By measuring the activity variations of these radionuclides at different sections along the loop, both the *in situ* monitoring of the corrosion attack depth of 316L SS and corrosion product transport and its precipitation in flowing NaCl-MgCl_2_ molten salt were achieved. While 316L SS was the focus of this study, the technique reported herein can be extended to other structural materials being used in a wide range of industrial applications.

## Introduction

Corrosion and degradation of structural materials in contact with high-temperature molten salts continues to be a limiting factor for developing sustainable energy systems including Generation IV molten salt reactors [^1^], thermal energy storage, and concentrated solar power plants [^2^,^3^]. Typically, high-temperature materials are self-passivating and therefore protected from corrosion induced degradation by forming passive oxide layers [^4^]. However, these oxide layers have a string tendency to dissolve in high-temperature molten salts, especially molten chloride and fluoride salts [^5^]. The absence of protective oxides results in the electrochemical dissolutions of thermodynamically susceptible alloying elements into the salt [5]. This leads to the thinning of structural components and to contamination of the salt itself. Additionally, the corrosion products can plate-out on the relatively cooler sections due to the change of chemical equilibrium constants as a function of temperature, resulting in fouling and restricted flow at heat exchangers [^6^,^7^]. It is of critical importance to systematically understand the corrosion mechanism in molten salt environments via *in situ* monitoring the corrosion process and degradation of materials, ensuring the materials’ compatibility with molten salts.

In recent years, significant efforts have been made to understand the corrosion mechanisms of different iron- and nickel-based alloys in high temperature molten salts in static conditions [^8^,^9^,^10^,^11^,^12^]. While static conditions are useful to down-select alloys based on their corrosion performances in well controlled environments, further investigations must be performed in non-isothermal flow conditions. Indeed, non-isothermal flow conditions induce thermal gradient corrosion as well as erosion if flow velocities are sufficiently high [^13^,^14^], both of which represent additional degradation mechanisms in molten salt environments. Structural material corrosion testing in flowing conditions dates back to the Aircraft Reactor Experiment, followed by the Molten Salt Reactor Experiment at Oak Ridge National Laboratory (ORNL) in the 1950s and 1960s [^15^,^16^,^17^], where corrosion studies were mainly carried out in closed natural circulation loops [13,14,^18^,^19^]. In many of these studies, samples were suspended in various sections of molten salt loops [^20^,^21^,^22^] followed by post-corrosion examination of the samples. Small-scale natural circulation loops, or microloops, were recently developed by TerraPower, where the loop pipe itself serves as the corrosion testing sample [^23^]. In all of the studies reported to date, corrosion and mass transport mechanism investigations are based on the post-test sample characterizations, such as microstructural examination and/or sample weight change measurements. Using these methods, the corrosion rates of different materials and precipitations of corrosion products can be evaluated at given exposure time. However, these approaches are not conducive to understanding the dynamic corrosion processes in flow conditions where elucidating corrosion kinetics under multiple driving forces requires in-situ techniques. Thus, it is crucial to develop an effective method to monitor *in situ* the corrosion and mass transport in a molten salt loop to unveil the dynamic corrosion mechanisms at play.

This gap in knowledge motivates the development of an experimental approach capable of *in situ* monitoring the corrosion and mass transfer in molten salt loop. Here we characterize the corrosion of 316L SS and the mass transport of corrosion products as a function of exposure time in a NaCl-MgCl_2_ eutectic molten salt natural circulation loop using a novel radionuclide tracing technique. This technique involved irradiating a thinned tube section later welded into the hot leg of the molten salt loop with a 16 MeV proton beam to produce radionuclides of interest including ${\;}^{51}\text{Cr}$ (t1/2 = 27.7 d, g = 320 keV), ${\;}^{52}\text{Mn}$ (t1/2 = 5.6 d, g = 744, 936, 1434 keV), and ${\;}^{56}\text{Co}$ (t1/2 = 77.2 d, g = 846, 1037, and 1238 keV) along the tube thickness. These radionuclides emit characteristic gamma rays upon decay which were detected, allowing location tracking as functions of exposure time. Thus, the material corrosion and elemental mass transport can be assessed *in situ* by monitoring the activity variations of the radionuclides at the irradiated tube location and/or at other parts of the loop. This *in situ* corrosion monitoring system was coupled with a molten salt microloop design [23]. By using the developed radionuclide tracing method, the *in situ* monitoring of the corrosion attack depth of 316L SS at the hot leg and corrosion product transport in the microloop has been achieved. To the authors’ knowledge, this study represents the first successful achievement to monitor the corrosion of materials *in situ* in molten salt flowing conditions and sheds new light on the corrosion mechanism of materials in non-isothermal flowing conditions.

## Results

### Molten salt microloop

**Figure 1:**
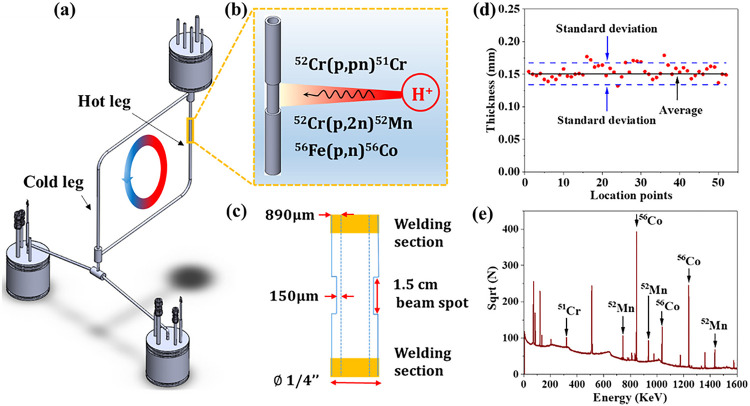
Radionuclide tracing system coupled with the molten salt microloop. (a) Schematic illustration of the 316L SS natural circulation microloop; (b) radionuclides generation by proton beam; (c) schematic of the thinned down tube section; (d) thickness of the thinned down tube section; (e) gamma spectrum obtained on the irradiated tube section by HPGe detector.

As an alloy for high temperature system, 316L SS was selected for the construction of the molten salt microloop. The loop itself served as the testing materials and no testing coupons were introduced in the loop. The loop was naturally circulated with the hot leg maintained at 620 °C while the coldest section stabilized at around 500 °C during operation. About 150 g of NaCl-MgCl_2_ eutectic salt (58.5 mol% NaCl-41.5 mol% MgCl_2_, melting point: 445 °C) provided by ORNL was loaded inside the loop and flowed counterclockwise from the hot leg to the cold leg, driven by the temperature gradient as shown in Figure 1(a). As mentioned in the methods section, the Transient Simulation Framework of Reconfigurable Models (TRANSFORM) code, developed at ORNL [^24^], was utilized in this study to model the natural circulation of the microloop. Using the power to heaters as inputs while the measured temperatures by multiple thermocouples around the loop as outputs, the molten salt flow rate was determined to be about 6.3 cm/s.

### Radionuclide generations and distributions

To produce the radionuclides, a small tube section (about 1.5 cm in length) at the mid-section of the hot leg of the microloop was irradiated from the outer surface with a 16 MeV proton beam (Figure 1(b)). Considering the original thickness of the tube (about 890 μm) is too thick for the proton beam to penetrate through and generate radionuclides at the salt/alloy interface, the tube section at the hot leg to be irradiated was thinned down to about 150 μm as shown schematically in Figure 1(c). Figure 1(d) displays the thickness of the thinned section measured at different locations by a Magna-Mike^®^ 8600 magnetic probe. As illustrated in the figure, the tube was thinned down relatively uniformly over the entire thinned section length. It is expected that the radionuclides ${\;}^{51}\text{Cr}$, ${\;}^{52}\text{Mn}$, and ${\;}^{56}\text{Co}$ will be produced along the tube thickness. These radionuclides emit characteristic gamma rays through their decay process, which were detected in the post-irradiated tube section and other sections around the loop by a High Purity Germanium (HPGe) detector, calibrated for energy and efficiency using an Americium-241 check source of known activity. Figure 1(e) shows a representative gamma ray spectrum obtained from the irradiated tube section after irradiation, where the peaks of ${\;}^{51}\text{Cr}$, ${\;}^{52}\text{Mn}$, and ${\;}^{56}\text{Co}$ are clearly displayed.

**Figure 2:**
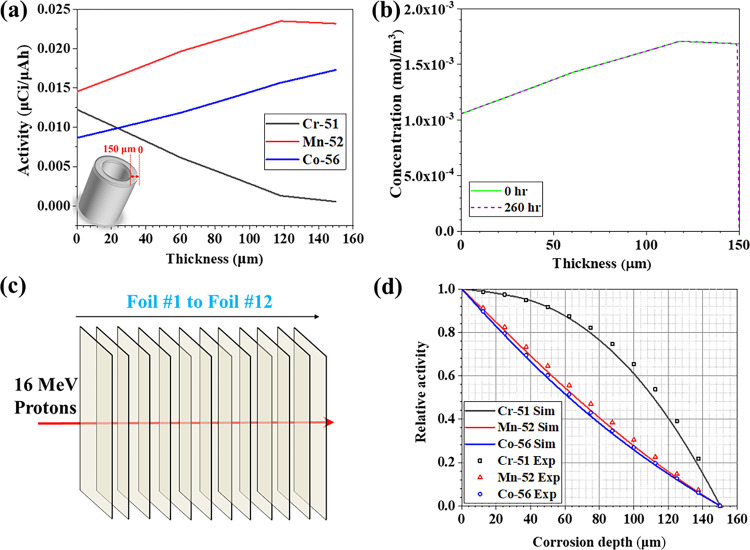
Acquisition of activity and relative activity profiles of radionuclides. (a) schematic of the irradiated tube section; (a) activity of radionuclides along the irradiated tube thickness; (b) concentration profiles of ${\;}^{52}\text{Mn}$ along the irradiated tube thickness before and after loop operation due to the elemental diffusion; (c) schematic of the irradiation of a stack of 12 slices of 316L SS foils; (d) relative activity profile of radionuclides at different recession depth.

The reaction rates for the formation of radionuclides vary as a function of the degrading energy of the proton beam, resulting in varying concentration of radionuclides along the tube thickness after irradiation. Based on the cross-sections of radionuclides production reaction as a function of proton beam energy [^25^], the radionuclide activity profile was determined as a function of the position in the sample using equation (1) [^26^]

(1) $$A=N\cdot \chi \cdot \sigma \cdot I\cdot \left(1-e^{-\lambda t}\right)$$

where A is the activity in Bq, N represents the number of target atoms per cm^3^, x is the distance travelled by the incident proton beam in cm, I is the beam current in μAh which was converted to protons/s hitting the target, σ is the reaction cross-section of interest in cm^2^ which was determined from semi-empirical predictions of the TALYS code [25], λ is the associated decay constant in s^−1^, and t is the time of exposure in s. As shown in the inset of Figure 2(a), the outer surface of the irradiated tube section was defined as the origin and the inner surface as the thickness of 150 μm. The modeled activity profiles in nCi/μAh are plotted in Figure 2(a) as a function of location in the irradiated 316L SS alloy sample. The results show that the activity of ${\;}^{52}\text{Mn}$ and ${\;}^{56}\text{Co}$ increase slightly with the tube thickness. On the contrary, the activity of ${\;}^{51}\text{Cr}$ sharply decreases with the thickness.

Radionuclides may also diffuse in the alloy due to the high operation temperature and elemental corrosion at the salt/alloy interface, altering the activity profiles described above during the *in situ* corrosion study. Based on the initial activity profiles as shown in Figure 2(a), the evolution of radionuclides can be modeled over time. Taking ${\;}^{52}\text{Mn}$ as an example, a simple diffusion model based on Fick’s law (equation (2)) can be used,

(2) $$\frac{\partial C}{\partial t}=D\frac{\partial^{2}C}{\partial x^{2}}$$

with D the diffusion coefficient of Mn (assumed to be 10^−19^ m^2^/s as reported by Smitll et al [^27^]). Using boundary conditions of no-flux at the tube outer surface and of zero Mn concentration at the salt/alloy interface, the concentration profile (which also represents the activity profile) of ${\;}^{52}\text{Mn}$ after 260 hours (i.e., the loop operation time) barely evolved at the hot leg as shown in Figure 2(b) (about 99.7% of the ${\;}^{52}\text{Mn}$ activity is still present after 260 hours). Consequently, the activity profile change by diffusion-induced radioisotope corrosion into the salt is likely negligible. Thus, the activity loss observed experimentally should be mainly induced by surface recession rather than by diffusion-induced corrosion. To assess surface recession, the “relative activity” was defined as the activity remaining in the tube after a certain thickness of the tube had recessed, assuming no thermally driven diffusion of radionuclides, divided by the initial radionuclide activity. Based on the calculated activity profile as displayed in Figure 2(a), the relative activity profiles of ${\;}^{51}\text{Cr}$, ${\;}^{52}\text{Mn}$, and ${\;}^{56}\text{Co}$ as a function of the recessed layer thickness can be derived as shown in Figure 2(d). Thus, one can theoretically measure the radionuclide activity remaining in the tube during exposure, calculate the relative activity, and derive the tube recession rate *in situ*. However, this framework relies on the exact relative activity profiles of these radionuclides. To validate these profiles, 12 slices of 316L SS foils with the thickness of 12.5 μm each were stacked together (overall thickness: 150 μm) and irradiated with 16 MeV protons as shown in Figure 2(c). The activity of each 316L SS foil was measured by a HPGe detector. Removing one slice of foil each time from foil #1 to foil #12 is an equivalent process of 12.5 μm of tube thickness being corroded or recessed. Through dividing the activity of the remaining foils after each removal by the total activity of the 12 slices of foils, the relative activity at the recessed depths of 12.5 μm, 25 μm, 37.5 μm, 50μm, 62.5μm, 75 μm, 87.5 μm, 100 μm, 112.5μm, 125 μm, 137.5 μm, and 150 μm were calculated, respectively. Figure 2(d) shows the comparison of the experimental relative activity data points obtained after the foil irradiation experiment with the modelled relative activity profile obtained by equation (1). Both results agree quite well, lending confidence that the relative activity measurements are a reliable indicator of corrosion induced surface recession rate.

### *In situ* corrosion monitoring

**Figure 3:**
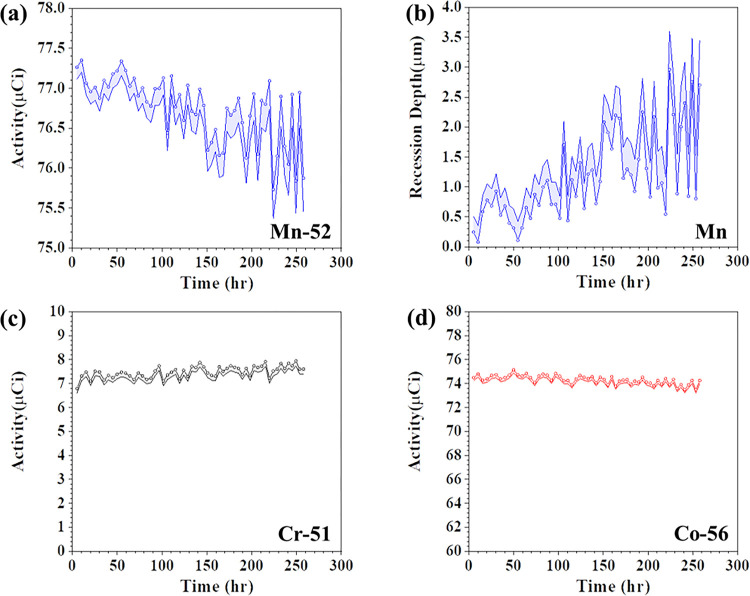
*In situ* corrosion monitoring of 316L SS in the molten salt loop by radionuclide tracing. (a) activity variation of ${\;}^{52}\text{Mn}$ with time during the loop operation process; (b) derived recession depth variations of Mn in 316L SS with time during the loop operation process; (c) activity variations of ${\;}^{51}\text{Cr}$ and (d) ${\;}^{56}\text{Co}$ with time during the loop operation process.

The molten salt microloop was naturally circulated for about 260 hours by maintaining the hot leg at 620 °C and the coldest section at around 500 °C, after which, the cold leg temperature reached values below the NaCl-MgCl_2_ melting point, resulting in loss of natural circulation. The irradiated tube gamma-ray spectra were acquired continuously with a same time interval during the natural circulation process using an Ametek Ortec ICS-P4 HPGe detector. By analyzing the full energy peaks of ${\;}^{51}\text{Cr}$, ${\;}^{52}\text{Mn}$, and ${\;}^{56}\text{Co}$ in the obtained gamma-ray spectra, the activity of these three radionuclides was derived as a function of time and results are displayed in Figure 3. Considering the existence of natural decay process of each radionuclide, the measured activity was decay-corrected to the end of bombardment (EOB) when each radionuclide was generated. Specifically, Figure 3(a) shows the decay-corrected activity of ${\;}^{52}\text{Mn}$ as a function of exposure time. It is observed that the activity decreases slightly as a function of exposure time, indicating a loss of ${\;}^{52}\text{Mn}$ from the tube due to molten salt corrosion. Comparing the activity of ${\;}^{52}\text{Mn}$ at the beginning and end of the loop operation, the activity loss of ${\;}^{52}\text{Mn}$ in the hot leg is about 2%. This is about an order of magnitude higher than the modeled diffusion-induced activity loss as discussed above, indicating the corrosion of Mn mainly results from surface recession. The relative activity of ${\;}^{52}\text{Mn}$ was determined through dividing the activity of ${\;}^{52}\text{Mn}$ shown in Figure 3(a) by its activity at EOB. Using Figure 2(d), the recession depth of Mn in 316L SS was extracted based on the determined relative activity and presented in Figure 3(b), and it is found to be about 3.5 μm at the end of loop operation.

The activity variations of ${\;}^{51}\text{Cr}$ and ${\;}^{56}\text{Co}$ as a function of exposure time are shown in Figure 3(c) and (d), but no obvious activity loss is observed. The activity of ${\;}^{51}\text{Cr}$ remains stable during the loop operation process, although Cr is thermodynamically susceptible to corrosion, and typically dissolves into molten salt during molten salt corrosion in the form of chromium divalent or trivalent ions [^28^]. This lack of Cr activity variation as a function of exposure time is attributed to the much lower concentration of the ${\;}^{51}\text{Cr}$, relative to ${\;}^{52}\text{Mn}$, at the inner diameter of the irradiated tube (see Figure 2(a)). Consequently, any ${\;}^{51}\text{Cr}$ activity loss induced by ${\;}^{51}\text{Cr}$ dissolution into the salt during the loop operation is beyond the sensitivity of the gamma-ray detector. The activity of ${\;}^{56}\text{Co}$ also remains relatively constant during exposure. This is expected since the standard Gibbs free energy of formation of CoCl_2_/Cl (=−238 KJ/mol) is much higher than the other main constituent elements of 316L SS such as Cr (=−277 KJ/mol) and Fe (=−279 KJ/mol) based on the thermodynamic database collected by HSC Chemistry 6.0 [^29^]. As a result, Co was less likely to corrode during the operation of the molten salt loop.

**Figure 4:**
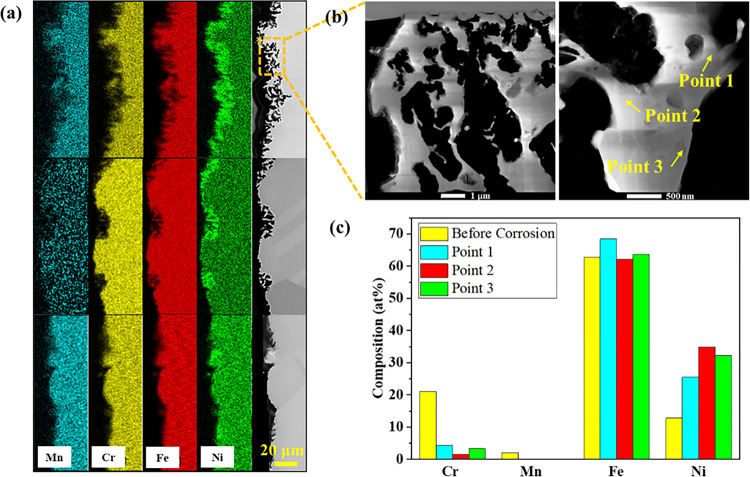
Characterizations of samples after flow loop corrosion testing. (a) SEM image of the tube cross section close to the irradiated section at the hot leg of the loop after loop operation; (b) STEM HAADF images of corroded layer; (c) STEM EDS point scans of the main elements’ compositions in the remnants of the 316L SS alloy.

Post corrosion material characterization was performed on different parts of the loop after corrosion testing. Figure 4(a) displays the SEM/EDS imaging of the cross section of the post-corroded tube from the hot leg, close to the irradiated section. A severe corrosion attack was observed at the salt/alloy interface with a surface morphology typically observed in flowing molten salt corrosion [^30^]. Slight dissolutions of Mn and Cr, and to a lesser extent Fe were observed, while Ni is relatively enriched at the interface. In addition, based on the SEM/EDS analyses taken at different locations, it appears that the corrosion occurring at the hot leg is relatively heterogeneous, as already observed in previous study [23]. The surface of the alloy does not recess homogeneously, likely because of heterogeneous dissolution of thermodynamically susceptible elements. Consequently, the surface morphology evolves and the pores, also called wormholes [^31^], are observed. In addition to the recessed layer, there are also regions exhibiting negligible corrosion. As shown in Figure 4(a), the depth at which pores are observed can be as high as 10 μm in some areas (see the top of the SEM image in Figure 4(a)), while corrosion at other areas was not clearly observed (see the bottom of the SEM image in Figure 4(a)). STEM characterization was further performed on the intense corrosion attack zone and results are shown in Figure 4(b). The results confirm that the corrosion proceeded via the preferential leaching of the thermodynamically unstable elements. Early studies [^32^,^33^] proposed that salt can infiltrate into the alloy subsurface regions and further corrode the alloy by dissolving the electrochemical susceptible elements. This is evidenced by the STEM-EDS point scans in this study showing that the Cr and Mn concentrations in the remnants of the alloy were significantly reduced as displayed in Figure 4(c).

**Figure 5:**
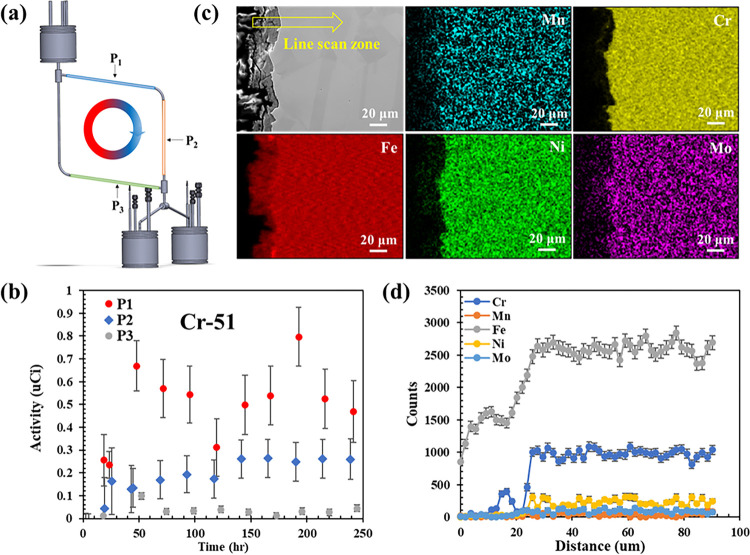
Transport and deposition of corrosion products along the molten salt loop. (a) Detecting points of the HPGe detector along the loop. (b) activity variations of ${\;}^{51}\text{Cr}$ at three different locations along the loop during its operation. (c) SEM/EDS imaging on the cross section of the tube section from cold leg. (d) EDS line scan on the marked zone shown in (c).

With the radionuclide tracing method, another ICS-P4 HPGe detector was utilized to measure the activity variations of radionuclides at three different locations: P_1_, P_2_, and P_3_ around the loop as displayed in Figure 5(a) during the loop operation. This measurement system allowed for the characterization of the transport and possible redeposition of the corrosion products. Figure 5(b) shows the activity variations of ${\;}^{51}\text{Cr}$ as function of exposure time at these three different locations. The activity levels of ${\;}^{51}\text{Cr}$ at these three locations are decreasing from P_1_ to P_3_. This result is likely due to the decrease in radionuclide concentration within the salt, resulting from the deposition of corrosion products along the loop. While the ${\;}^{51}\text{Cr}$ activity loss was not detectable in the hot leg, ${\;}^{51}\text{Cr}$ activity was detected in the salt. However, it should be noted that the level of the detected ${\;}^{51}\text{Cr}$ activity in the salt is much lower than that in the irradiated tube (by about one order of magnitude), which is likely the reason why the activity loss of ${\;}^{51}\text{Cr}$ was not statistically detected in the tube, i.e., the activity loss is within the detection noise of the activity in the irradiated tube. Another interesting phenomenon is the lack of ${\;}^{51}\text{Cr}$ activity in the salt (and on the tube) at P_3_, right before entering the hot leg. This means that all the Cr dissolved from the hot leg redeposited along the loop within the same cycle. Basically, there is no recirculation of activated corrosion products in the loop. This is consistent with findings of a previous study that the overall corrosion in the hot section equals to the precipitation in the cold section, over an entire closed loop [^34^]. This is also evidenced by the increased activity of ${\;}^{51}\text{Cr}$ at P_2_ as a function of exposure time, which results from the deposition of Cr at that location, in addition to the activity from the flowing salt. To further verify the depositions of corrosion products at the cold leg, SEM/EDS analysis was performed on the cross section of a tube section from the cold leg after corrosion testing and the results are shown in Figure 5(c). A deposited layer is clearly visible at the alloy/salt interface. EDS mapping and line scan (Figure 5(d)) illustrate that the deposited layer is rich in Fe, with little Cr. The Cr deposition at the cold leg observed by post-test material characterization is qualitatively consistent with the *in situ* result obtained by radionuclide tracing.

## Discussion

**Figure 6:**
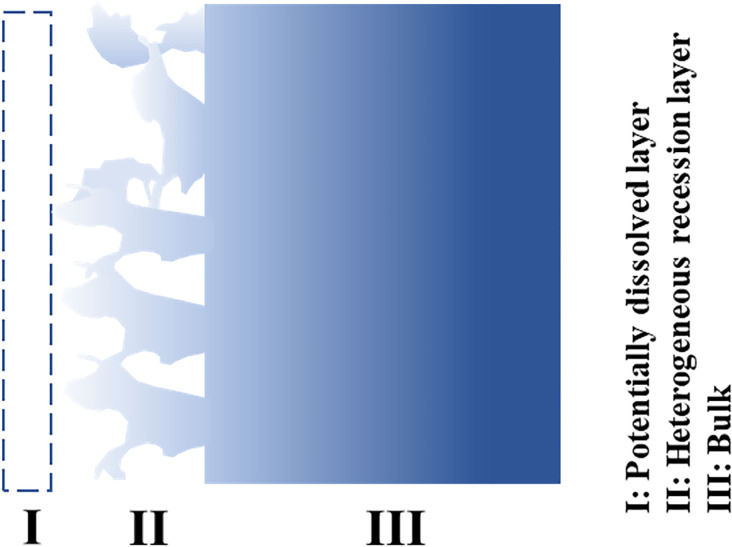
Schematic of the corrosion attack of alloy in molten salts.

This study involves the development of a novel radionuclide tracing technique for *in situ* corrosion and mass transport monitoring in molten salt loop and has been used for studying the corrosion mechanism of 316L SS in flowing NaCl-MgCl_2_ eutectic molten salt. The results of the present study demonstrate that radionuclide tracing is a viable method for material corrosion monitoring, especially in molten salts in which material loss is involved. The present work represents the first time to achieve the *in situ* monitoring of materials corrosion and mass transport of corrosion products in molten salt environment. The corrosion attack of the 316L SS alloy in this study can be schematically divided into three zones as displayed in Figure 6: (I) the potentially dissolved layer (i.e., surface recession) which cannot be detected by typical material characterization methods such as SEM/EDS/TEM; (II) the heterogenous surface recession layer with bulk material partially dissolved; and (III) bulk material. Elemental composition gradient might exist in zone (II) and (III) because of diffusion as displayed in Figure 6. However, based on the results from the *in situ* detection as discussed above and the post corrosion material characterization by SEM/EDS as shown in Figure 4(a), there are no significant corrosion induced diffusion profiles within the corroded layer. It is worth noting that the radionuclide tracing method developed in this study measures the overall corrosion rather than localized corrosion as observed by post-corrosion characterization. Therefore, the surface recession depth of 316L SS by flowing NaCl-MgCl_2_ eutectic molten salt measured by this method is an overall evaluation of the corrosion behavior of the entire irradiated tube section at the hot leg. The surface recession depth determined from activity loss is averaged over the entire probed tube surface although it is clear from post-corrosion microscopy characterizations that the attack is relatively heterogeneous with a complex surface morphology development. The in-situ radionuclide detection method accounts for the fully dissolved layer (zone I in Figure 6), the heterogenous surface recession layer (zone II in Figure 6), and the barely attacked substrate. Consequently, while the surface recession depth obtained by the radionuclide tracing method is about 3.5 μm, the corrosion attack can be as deep as 10 μm locally (as displayed in Figure 4(a)).

The corresponding 316L SS weight loss from a surface recession depth of 3.5 μm is about 2.8 mg/cm^2^. This is about 3 to 4 times higher than the 316L SS weight loss of about 0.8 mg/cm^2^ observed in static NaCl-MgCl_2_ eutectic salt at 600 °C for 240 hours [^35^]. However, the salt volume of ~150 g used in our natural circulation loop is also about 3 times of the salt volume (60 g) in the static corrosion experiment [33]. In addition, considering the influence of flow on corrosion and the slight difference of the experimental conditions of temperature and corrosion testing time, these two corrosion results are relatively comparable. This is an indirect comparison between the corrosion of static and flow conditions, a direct comparison will be needed in the future to study the corrosion behaviors under static and flow by setting same experimental conditions. A corrosion study of 316L SS in NaCl-MgCl_2_ molten salt natural circulation loop has been similarly performed for 1000 hours [23], but the 316L SS corrosion performance was only evaluated by post material characterization methods (i.e., at the end of loop operation). It is worth noting that the corrosion attack depth of 316L SS at the same location in the loop after 1000 hours of natural circulation was found to be about 20 μm, which is in qualitative agreement with the results reported in this study for 260 hours of exposure, validating the corrosion results reported in this study.

The radionuclide tracing method developed in this study essentially is a technique to measure the variation of activity occurring by material’s loss. Cr and Mn are two main constituent elements for high-temperature (>700 °C) alloys and are susceptible to dissolution in molten salts. Therefore, ${\;}^{51}\text{Cr}$ and ${\;}^{52}\text{Mn}$ could be used for the *in situ* corrosion and mass transport monitoring of high temperature alloys when exposed to molten salt environments. ${\;}^{56}\text{Co}$ is also produced via nuclear reactions such as ${\;}^{56}\text{Fe}(\text{p},\text{n}){\;}^{56}\text{Co}$, although Co is not an element of interest to measure materials’ corrosion, since it is relatively thermodynamically stable and should experience little dissolution in molten salts. The original distribution of the generated radionuclide tracer in the material is also crucial for the *in situ* corrosion monitoring. Unfortunately, in our study of 316L SS, the concentration of ${\;}^{51}\text{Cr}$ at the tube inner diameter is too low and the corresponding activity loss was not detectable. A higher concentration of radionuclide tracers distributed at the salt/alloy interface, would make the radionuclide tracing method more sensitive to *in situ* corrosion monitoring of ${\;}^{51}\text{Cr}$. On the other hand, a few micrometers of Mn depletion can be detected through the activity measurement of ${\;}^{52}\text{Mn}$ because of the relatively high concentration of ${\;}^{52}\text{Mn}$ at exposed surface. However, the overall concentration of dissolved ${\;}^{52}\text{Mn}$ is relatively small and coupled to a relatively short half-life, resulted in undetectable activity level of the dissolved ${\;}^{52}\text{Mn}$ in NaCl-MgCl_2_ molten salt. Consequently, the mass transport study of Mn in the loop was not feasible. As a surrogate, the detectable ${\;}^{51}\text{Cr}$ in the loop can be used to study the mass transport of corrosion products although it is not feasible for *in situ* measurement of depletion depth. The complementarity of the activity measurements of multiple radionuclides could be an ideal way for the *in situ* corrosion evaluation and mass transport monitoring of corrosion products in molten salts.

## Methods

### Salt preparations

The NaCl-MgCl_2_ eutectic salt used in this study was prepared with 58.5 mol% anhydrous NaCl salt and 41.5 mol% anhydrous MgCl_2_ salt. The salt was homogenously mixed and purified by ORNL. The trace impurities of the NaCl-MgCl_2_ eutectic salt mixture were identified by ICP-MS analysis in which the most prominent impurity elements and the corresponded concentrations were 0.79 ppm Li, 5.44 ppm S, 15.7 ppm K, 6.99 ppm Ca, 0.03 ppm Cr, 0.01 ppm Mn, 0.13 ppm Fe, 0.23 ppm Ni.

### Molten salt microloop design and operation

The molten salt microloop built in this study was built with 316L SS based on the design by TerraPower [23]. The nominal chemical composition of 316L SS is given in Table S1 in the supplementary document. While the schematic of the microloop and its auxiliary equipment is shown in Figure S1 in the supplementary document. The loop itself is the corrosion testing material to be investigated. The dimensions of the whole loop frame are 32” × 32” × 32” and the loop body is a 9.9”×12.3” parallelogram. The tube diameter of the loop is 0.25” with a wall thickness of 0.035”. The loop is micro, requiring only two, 96 cm^3^ batches of salt for continuous operation. The molten salt flow in the microloop is established through natural circulation by setting up a hot leg and a cold leg sections, as detailed in [23]. In this design, a temperature gradient across the harp shaped portion of the microloop generates salt density gradients resulting in buoyant forces that drive natural circulation of the molten salt. There are three tanks in the microloop as shown in Figure S1 in the supplementary document. The bottom of the microloop has a flush salt tank and a primary salt tank. The flush salt was stored in the flush salt tank to “clean” the residual weld oxides and debris from the microloop construction before the test, while the primary salt was stored in the primary tank for the actual test. The surge tank at the top is designed for safety to allow for excess molten salt volume to expand, if necessary, without pressure build-up. The microloop’s main body was wrapped with nichrome heating wire to heat up different sections of the loop to their target temperatures and initiate natural circulation. Thermocouples were welded onto the tube outer wall surface at different sections of the loop to monitor the temperature. The two legs at the bottom of the microloop (see Figure S1 in the supplementary document) were equipped with ceramic heaters serving as freeze valves. To fill molten salt into the loop, the salt in the primary salt tank (or flush salt tank) was first melted and then primed into the loop’s main body by argon gas pressure through a gas line connected to each tank. The power of the ceramic heaters serving as the freeze valves was switched to a temperature lower than the salt melting point immediately after the molten salt is primed into the loop. Following this, the molten salt in that section froze, blocking the tube, such that the molten salt in the loop would not flow back into the tank. Once the main body of the loop was filled with molten salt, natural circulation was initiated by setting a temperature gradient along the loop using nichrome heating wire and monitoring the thermocouples at different sections of the loop. After the natural circulation was initiated, the loop was operated continuously with ~5 psig argon cover gas to avoid the possible uptake of atmospheric air into the loop through microleaks.

### Radionuclide production

To enable radionuclide tracing, a small section of tube must be irradiated, activating the tube’s inner diameter contacting the salt. Thus, the proton beam used to generate radionuclide had to be of sufficient energy and intensity at the inner tube location to overcome the activation cross-section energy thresholds and obtain relatively large reaction rates. However, the 16 MeV proton beam energy available for this experiment was too low to generate a useful activation profile for radionuclides of interest across the entire tube thickness (~890 μm). A thickness of about 150 μm would generate relatively significant radionuclide concentration at the inner surface of the tube to enable gamma-ray detection. A tube section about 15 cm in length was cut from the hot leg of the microloop. The central part of that section (~1.5 cm in length, corresponding to beam spot size for irradiation) was thinned down to about 150 μm using a lathe. To irradiate the thinned 316L SS section, a custom target holder and slit collimator was designed and fabricated (see Figure S2 in the supplementary document). Before irradiation, the tube was placed into the target holder and sealed at the two ends by Swagelok fittings. By adjusting the position of the tube, the thinned 1.5 cm section of the tube can be aligned below the slit of the collimator. The target holder was connected to the cyclotron beam port by a KF flange (see Figure S2 in the supplementary document). The two Swagelok fitted ends were connected to a water line outside the cyclotron, allowing internal water cooling to the tube and stop the ion flux. The tube was irradiated with 20 μAh beam fluence using a General Electric PETtrace cyclotron located at the Wisconsin Institutes for Medical Research. The displacement per atom (DPA) induced by the beam is quite small, and the radiation damage on the alloy could be neglected. The radionuclides of interest as tracers for *in situ* corrosion monitoring in the microloop are ${\;}^{51}Cr,\;{\;}^{52}Mn$, and ${\;}^{56}Co$. ${\;}^{51}\text{Cr}$ was produced via the nuclear reactions shown in equation (3)

(3) $$\begin{array}{c} {\;}^{52}\text{Cr}{\left(\text{p},\text{pn}\right)}^{51}\text{Cr}\\ {\;}^{52}\text{Cr}{\left(\text{p},2\text{n}\right)}^{51}\text{Mn}\to (\text{decay})\to^{51}\text{Cr}\\ {\;}^{54}\text{Fe}{\left(\text{p},\alpha \right)}^{51}\text{Mn}\to (\text{decay})\to^{51}\text{Cr} \end{array}$$

while ${\;}^{52}\text{Mn}$ was produced through the reaction (4).


(4) $${\;}^{52}\text{Cr}{\left(\text{p},\text{n}\right)}^{52}\text{Mn}$$

${\;}^{56}Co$ was produced through the reactions shown in equation (5)

(5) $$\begin{array}{c} {\;}^{56}\text{Fe}{\left(\text{p},\text{n}\right)}^{56}\text{Co}\\ {\;}^{57}\text{Fe}{\left(\text{p},2\text{n}\right)}^{56}\text{Co} \end{array}$$

Although there are a few characteristic gamma emissions with the generated radionuclides ${\;}^{52}Mn$ and ${\;}^{56}Co$, the gamma emissions at 936 keV for ${\;}^{52}Mn$ while 1037 keV for ${\;}^{56}Co$ were selected for their quantifications to avoid the interference from the nearby signals.

### *In situ* corrosion and mass transport monitoring system

The 15 cm 316L SS tube containing the thinned and irradiated section of around 1.5 cm in length at its middle was welded back to the microloop using an orbital welding technique. Before welding, the potential influence of heat affected/fusion zone induced by welding on the corrosion and irradiation responses of the tube was characterized. This was achieved by performing orbital welding on prototypical 316L SS tube samples (see Figure S3 in the supplementary document). The welded tube section was cut axially, mounted in epoxy, polished using SiC paper with different grit size from 320 to 1200 grits, etched with aqua regia, and then analyzed by optical microscopy (see Figure S3 in the supplementary document). Results showed that the total length of fusion zone was about 2.5 cm, while the heat affected zone was not detectable at this magnification. To avoid the potential effects of the weld-induced microstructure on the corrosion of the proton activated section, the total section of the tube used was 15 cm, meaning the proton activated section was about 6.75 cm away from the weld section and would not be affected.

A pressure test of the microloop was performed to confirm the absence of leaks after welding the irradiated tube section back to the hot leg of the loop. Then, the nichrome heating wire and insulation material were rewrapped back on the tube section. The salt loading of the microloop was performed inside an inert glovebox filled with argon gas (O_2_<2ppm, H_2_O<2 ppm) since NaCl-MgCl_2_ salt are hygroscopic and very sensitive to moisture and oxygen in the air. Once the salt was loaded into the tank, the microloop was sealed by Swagelok fitting caps and pressurized with ~5 psig argon gas. The loop was moved out from the glovebox while the pressurized argon gas aids in preventing uptake of atmospheric air and moisture through potential microleaks.

The natural circulation of the microloop was initialized by setting up a temperature gradient. Once the natural circulation of the molten salt was established, the molten salt started to corrode the inner surface of the tube and the thermodynamically unstable elements (including radionuclides) started dissolving into molten salt. The radionuclides produced in the irradiated tube as a part of the loop would decay by emitting gamma rays. The *in situ* corrosion monitoring of the loop was achieved through measuring the activity variations of different radionuclides as functions of time and location by two Ametek Ortec ICS-P4 HPGe detectors. One HPGe detector faced the irradiated tube section at the hot leg of the microloop to monitor tube corrosion and the other HPGe detector was used to measure the activity variations of radionuclides at different locations around the loop (see Figure S4 in the supplementary document), allowing the characterization of the transport and redeposition of the corrosion products. To mitigate the interference of gamma rays from other locations/directions on the HPGe detectors, lead shields were installed in the system (see Figure S4 in the supplementary document).

### Material Characterization

Scanning electron microscope (SEM) coupled with energy-dispersive X-ray spectroscopy (EDS) was performed at the Wisconsin Center for Nanoscale Technologies to characterize the samples after corrosion experiment. Zeiss LEO 1530 device equipped with an energy dispersive x-ray spectrometer and Pathfinder software was used for the SEM/EDS analysis. Samples for the scanning transmission electron microscope (STEM) analysis were prepared using a standard lift-out technique by an FEI Helios PFIB G4 FIB/FESEM Focused Ion Beam (FIB) instrument in the Materials Science Center at the University of Wisconsin-Madison. The high-angle annular dark-field scanning transmission electron microscopy (HAADF-STEM) images and EDS in an FEI Titan G2 80–200 (S)TEM equipped with the EDS detector. The microscope was operated at an acceleration voltage of 200 kV, with a probe current of approximately 300 pA and a probe convergence angle of 21 mrad. A dwell time of 30 s was used for each point scan to achieve good statistics. Simultaneous HAADF and EDS acquisition were performed using the Bruker Esprit software. The cross section of each tube to be characterized was mounted with epoxy, ground with SiC abrasive papers of different grit sizes up to 1200 grit, and then polished on the polishing pads by 3 μm, 1 μm diamond suspensions, and 0.04 μm colloidal silica suspension.

### Loop circulation rate modelling

Transient Simulation Framework of Reconfigurable Models (TRANSFORM) code developed at Oak Ridge National Laboratory [24] was utilized in this study to model the natural circulation of the microloop. In the simulation (see Figure S5 in the Supplementary Document), each pipe was modelled as a single connected loop, cold leg section was modelled as pipe with a single heat transfer surface on which a circulation boundary condition was imposed, the heaters in the loop were modelled with internal heat generation equivalent to the output data obtained from the loop operation process. Through adjusting the molten salt flow rate in the PID controller added in the TRANSFORM model to maintain the temperature at different locations of the loop to best match the measured thermocouple data in the loop operation (see Figure S6 in the Supplementary Document), the molten salt flow rate during the loop operation was determined. More detailed information regarding the simulation process can be found in Refs [22,24].
